# Validation of Cognitive Load During Inquiry-Based Learning With Multimedia Scaffolds Using Subjective Measurement and Eye Movements

**DOI:** 10.3389/fpsyg.2021.703857

**Published:** 2021-08-31

**Authors:** Marit Kastaun, Monique Meier, Stefan Küchemann, Jochen Kuhn

**Affiliations:** ^1^Department of Biology Education, Institute for Biology, Universität Kassel, Kassel, Germany; ^2^Physics Education Research Group, Department of Physics, Technische Universität Kaiserslautern, Kaiserslautern, Germany

**Keywords:** cognitive load, cognitive abilities, representation preference, scaffolding, eye tracking, scientific inquiry

## Abstract

Subject-method barriers and cognitive load (CL) of students have a particular importance in the complex learning process of scientific inquiry. In this work, we investigate the valid measurement of CL as well as different scaffolds to reduce it during experimentation. Specifically, we examine the validity of a subjective measurement instrument to assess CL [in extraneous cognitive load (ECL), intrinsic cognitive load, and germane cognitive load (GCL)] during the use of multimedia scaffolds in the *planning* phase of the scientific inquiry process based on a theoretical framework of the CL theory. The validity is analyzed by investigating possible relationships between causal (e.g., cognitive abilities) and assessment (e.g., eye-tracking metrics) factors in relation to the obtained test scores of the adapted subjective measurement instrument. The study aims to elucidate possible relationships of causal factors that have not yet been adequately investigated in relation to CL. Furthermore, a possible, still inconclusive convergence between subjective test scores on CL and objectively measured indicators will be tested using different eye-tracking metrics. In two studies (*n*=250), 9th and 11th grade students experimentally investigated a biological phenomenon. At the beginning of the *planning* phase, students selected one of four multimedia scaffolds using a tablet (Study I: *n*=181) or a computer with a stationary eye-tracking device (Study II: *n*=69). The subjective cognitive load was measured *via* self-reports using a standardized questionnaire. Additionally, we recorded students’ gaze data during learning with the scaffolds as objective measurements. Besides the causal factors of cognitive-visual and verbal abilities, reading skills and spatial abilities were quantified using established test instruments and the learners indicated their representation preference by selecting the scaffolds. The results show that CL decreases substantially with higher grade level. Regarding the causal factors, we observed that cognitive-visual and verbal abilities have a significant influence on the ECL and GCL in contrast to reading skills. Additionally, there is a correlation between the representation preference and different types of CL. Concerning the objective measurement data, we found that the absolute fixation number is predictive for the ECL. The results are discussed in the context of the overall methodological research goal and the theoretical framework of CL.

## Introduction

Cognitive load (CL) is a theoretical, psychological construct that describes the individual loads of learners during the processing, construction, and memorizing of (new) information (Sweller et al., 2019). Within the Cognitive Load Theory (CLT), one of many basic assumptions is that only a limited number of information elements can be processed simultaneously due to the limited working memory capacity (Paas et al., 2010). Therefore, the processing of information that is relevant to learning should be optimized and loads irrelevant to learning are to be minimized (among others van Merriënboer et al., 2006; Paas and van Merriënboer, 2020). Another assumption claims the processing of information in two different channels – the verbal and the visual channel (dual channel assumption; Skuballa et al., 2019; Sweller et al., 2019; Scheiter et al., 2020). Furthermore, it is assumed that CL is composed of three distinct categories – intrinsic (ICL), extraneous (ECL), and germane cognitive load (GCL; among others Sweller, 1994; Zu et al., 2020). ICL is the cognitive load associated with high or low element interactivity during the processing of information/learning material (Sweller et al., 2011, 2019). Accordingly, the degree of interactivity between essential information elements required for learning determines the ICL. If the element interactivity is low, only a few elements are processed by the learner in working memory at the same time. If the element interactivity increases, the load on working memory also rises (Sweller et al., 2011). In contrast, ECL includes the cognitive load generated by suboptimal instructional design. Consequently, the learner needs to spend more cognitive resources on irrelevant or poorly constructed information during task performance or information processing than on the actual task solution (Sweller et al., 2019). The ECL and ICL together determine the total CL in the working memory by the learner. They are additively related because the resources for processing come from the same working memory pool (Sweller et al., 2011). The third category of cognitive load is GCL. This is expended by the learner to construct schemas and form mental models (Paas et al., 2003; Sweller et al., 2019), and it is therefore often described as the load that generates learning. In recent years, the study of CL has become increasingly important. To test and consequently optimize instructional designs or as a control variable for, e.g., self-regulated learning processes, such as inquiry-based learning (see “Environment (E) & Task (T) as causal factor – scaffolding & inquiry learning”; Kaiser et al., 2018), the valid measurement of CL represents a central goal of educational and psychological research (among others Kirschner et al., 2006; Minkley et al., 2018; Sweller et al., 2019). Using subjective self-assessments (Cierniak et al., 2009b; Leppink et al., 2013; Klepsch et al., 2017; Krell, 2017) or objective measures as indicators of CL, such as heart rate (Paas and van Merriënboer, 1994; Minkley et al., 2021), pupil dilation (Chen and Epps, 2014; Huh et al., 2019), blink rate (Chen and Epps, 2014), or gaze behavior (Korbach et al., 2018; Zu et al., 2020), different approaches to measuring CL have been investigated. Possible relationships and convergences between subjective and objective measurement tools for identifying CL are also coming more into the focus of research (among others Minkley et al., 2021). However, the use of subjective measurement instruments by self-assessment is still an established way to assess CL based on a learning task or instructional learning approach (Cierniak et al., 2009b; Leppink et al., 2013; Klepsch et al., 2017; Krell, 2017; Zu et al., 2020; Thees et al., 2021). Although the subjective measurement approach is controversial in research regarding valid measurement of CL (de Jong, 2010; Kirschner et al., 2011; Sweller et al., 2011), studies, such as the one by Klepsch et al. (2017), demonstrate a proven measurement accuracy. Other studies also reveal that measuring the three types of CL can pose different difficulties (DeLeeuw and Mayer, 2008; Ayres, 2018). First, it is unclear how, for example, the different CLs (ICL, GCL, and ECL) can be affected by the wording of items, since variations of the items lead to different results in performance and CL (Sweller et al., 2011; Klepsch and Seufert, 2021). Second, besides the wording, it is uncertain whether learners are able to assess their competences in relation to the differentiated items (ICL; GCL; and ECL; Sweller et al., 2011). Nevertheless, approaches to measure the three types of CL *via* self-assessments, e.g., *via* the design of controlled conditions (among others Sweller et al., 2011; Klepsch and Seufert, 2021), suggest that a categorical differentiation and measurement of the individual CLs are possible. However, Thees et al. (2021) also showed that relying on established subjective instruments to measure CL may lead to various difficulties. Composing a subjective measurement instrument from established items could lead to a more valid measurement of the three types of cognitive load (ECL, GCL, and ICL). However, these established self-assessments have mostly been used with university students, whereas research on subjective instruments measuring the different types of CL for middle-school students in instructional teaching-learning settings has been scarce (e.g., van de Weijer-Bergsma and van der Ven, 2021). Therefore, the focus of this research is to design and test a self-assessment questionnaire to measure the three types of CL for 9th and 11th grade students. To examine a possible valid measurement in combination with a convergence between subjective and objective measurement instruments of CL, various methods from validity research can be used. Consistent with the Standards for Educational and Psychological Testing (AERA et al., 2014), more recent validity approaches open possible opportunities for proving evidence and convergence *via* argumentative, theory-based test score interpretations (Messick, 1995; Kane, 2006, 2013). According to Kane (2013, p. 13), validity is “an integrated evaluation judgment of the degree to which empirical evidence and theoretical rationales support the adequacy and appropriateness of inferences and actions based on test scores or other modes of assessment”. Based on the validation framework of Cronbach and Meehl (1955), the Argument-Based Approach to Validation follows the examination of test score interpretation. This consists of making different validity assumptions from an interpretation-usage argument and testing them using different methods as well as relating them back to the existing theories (Kane, 2013). Hence, validity investigations do not represent a mere testing of formalized theories. They rather show the establishment and testing of coherent and plausible chains of reasoning that examine and explain the results of a measurement instrument in a theory- and evidence-based manner. Within these chains of reasoning, other validity criteria should be integrated and the test score should be examined from different perspectives (Messick, 1995; Kane, 2013). Regarding the validity testing of a subjective instrument to measure the three types of CL in this study (ICL, ECL, and GCL), the theory-based analysis of possible influences on CL and interactions within the learning environment needs to be investigated (causal factors: environment, task, and learner). The identification of possible relationships between these causal factors and the occurring ICL, ECL, and GCL (assessment factors) when using multimedia scaffolds is therefore required first. Then, a possible convergence between the results of the subjective instrument and objective indicators of CL will be examined in more detail. Therefore, the theoretical assumptions between the causal and the assessment factors will be presented first in order to deduct assumptions with regard to the validation of the measurement instrument that will be tested within the performed studies.

## Construct of Cognitive Load with Causal and Assessment Factors

**Figure 1:**
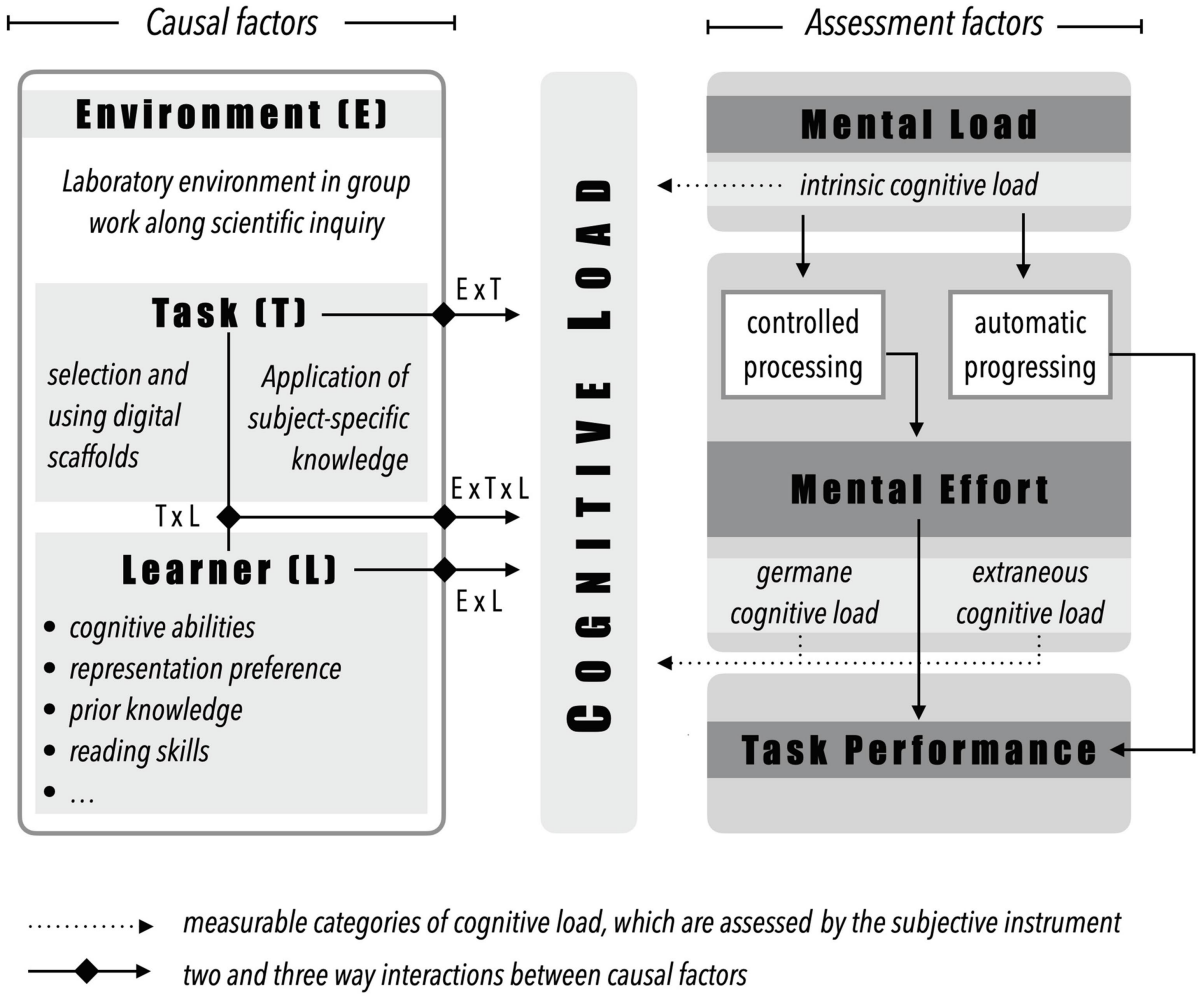
Construct of cognitive load with for the study-specific causal and assessment factors (adapted from Choi et al., 2014).

The distinction between causal and assessment factors in relation to CL was described by Paas and van Merriënboer (1994) based on study results in the context of problem solving. Choi et al. (2014) extended this theory-based construct of CL (Figure 1). In addition to the influencing factors of the task (task: e.g., complexity and format) and learner-specific characteristics (learner: e.g., cognitive abilities or prior knowledge), the model was extended by the factor of learning environment. Like the task as well as the learner characteristics, the environment can influence the load that occurs, e.g., physical conditions. The causal factors are in close interaction with each other which can significantly influence the expression of individual CL (Choi et al., 2014; Paas and van Merriënboer, 2020). The interaction between the causal factors can be seen, for example, between the task and the characteristics of the learner. Thus, different studies show the close interaction among prior knowledge and task complexity or task success (expertise-reversal effect: Cierniak et al., 2009a; Kalyuga, 2013).

Mental load, mental effort, and task performance are the assessment factors by which individual CL can be judged in relation to, for example, mastering a task or instruction. Both mental load and mental effort generally describe the CL that must be exerted by the learner to complete a given task. Mental load is the term used to summarize the subject-independent CL that relates solely to the characteristics of the task (Paas and van Merriënboer, 1994; Choi et al., 2014). Therefore, mental load is equivalent to the construct of ICL (e.g., Minkley et al., 2021). In contrast, mental effort describes the loads “which refers to the amount of capacity or resources that is actually allocated by the learner to accommodate the task demands” (Choi et al., 2014, p. 228). Mental effort therefore refers to the cognitive resources that are used during the specific problem solving/task and can accordingly be equated with GCL and ECL (e.g., Paas and van Merriënboer, 1994; Choi et al., 2014; Krell, 2017). Regardless of the classification of the individual types of CL, these are related to the causal factors that can influence both task performance and the CL of the learner. The level of prior knowledge (L), for example, has an influence on the expression of the ICL (see “Prior Knowledge”), whereas the ECL is considered to be mainly dependent on the instructional design (T). Both types of loads are perceived by the learner as rather passive (Klepsch and Seufert, 2021). The GCL, on the other hand, represents the perceived by the learner as rather active load (Seufert, 2018). The assessment factors can be measured by subjective (self-assessment) and objective (for example, heart rate) instruments as an indicator for CL (see “Introduction” and Figure 1). Subjective instruments, such as the ones of Zu et al. (2020); Klepsch et al. (2017); Krell (2017); or Leppink et al. (2013), have the benefit that they can be easily used *via* paper-pencil questionnaires within the intervention. However, contrary to objective measures, such as Heart Rate or Gaze Behavior, they depend on the self-assessment competences of learners/participants who assess and report their own load based on the item formulations (see “Introduction”). The objective instruments provide another way to measure CL. Although they are technical and costly, objective measurement methods are increasingly used. Although a valid measurement of CL cannot yet be granted, various studies show close correlations between subjective and objective measurement of CL categories (Korbach et al., 2018; Solhjoo et al., 2019; Minkley et al., 2021). Eye-movement measurements represent one research approach to investigate CL during learning with visual (and auditory) material or problem solving. Thus, different metrics, such as fixations duration (Zu et al., 2020) or transitions (Korbach et al., 2018), are used to derive learners’ CL. Previous work on the relationship of students’ gaze data and their subjective CL ratings demonstrated that certain eye-tracking metrics discriminated between the three types of CL (Zu et al., 2020). Zu et al. (2020) found that there is a significant relation of the mean fixation duration of students with low prior knowledge in a physics context and the ECL. Whereas the ICL significantly relates to the transitions between an animation and a text, the GCL is linked to the dwell time on an animation. These results in the context of electric motors were observed for students with low prior knowledge and they were independent on the working memory capacity.

### Environment (E) & Task (T) as Causal Factor – Scaffolding & Inquiry Learning

As shown in Figure 1, the learning environment and task are causal factors affecting CL. As Choi et al. (2014) noted, these two factors usually cannot be clearly separated in (terms of) practical implementation which also becomes clear by looking at the learning environment and the task in our study. In science education, inquiry-based learning is an approach to promote scientific reasoning as an integral part of science education (e.g., OECD, 2007). Within inquiry-based learning, learners are asked to actively apply different facets of knowledge to investigate a scientific phenomenon along the different phases of the scientific knowledge process (Kind and Osborne, 2017; de Jong, 2019). Through the generation of a research question and testable hypotheses, the planning of a scientific investigation to the execution and interpretation of the experimentally obtained results, learners actively use their content-related knowledge as well as their methodological skills (inquiry skills/scientific reasoning skills) to investigate the phenomenon/problem. Studies show that this problem-solving process (open inquiry learning: Bell et al., 2005), which is usually self-regulated and cooperative, has higher learning effects and can contribute better to long-term learning of scientific skills than direct forms of instruction (Alfieri et al., 2011; Furtak et al., 2012). However, other studies also suggest that learning along the scientific inquiry process can cause student-specific problems, as the complexity and openness of the learning process exceed the individual capacity of working memory (Leutner et al., 2005; Kirschner et al., 2006). As a result, supporting methods, such as protocol sheets provided with tasks, or the use of scaffolds, such as prompts and feedback, can minimize these occurring loads (Hmelo-Silver et al., 2007) and support the learner in his or her inquiry-based learning process (guided inquiry learning: Bell et al., 2005). Studies show, among other things, that short, direct prompts do not hinder the constructivist learning method of inquiry-based learning. However, depending on the design and the use, they can minimize individual CLs (e.g., Kirschner et al., 2006). Based on this, different approaches to scaffolding have been explored empirically (e.g., Arnold et al., 2014; Kaiser and Mayer, 2019; Meier and Kastaun, 2021). Using digital media, scaffolds can enable individual learning in a variety of forms.

In addition to prior knowledge and motivational factors, learners bring in further characteristics that offer starting points for differentiation. Especially when dealing with multimedia-based representation combinations (e.g., video or animation), cognitive characteristics, such as learning preferences for visual or verbal learning material (representation preference; Mayer and Massa, 2003), spatial ability (Höffler and Leutner, 2011), or cognitive style (Höffler and Schwartz, 2011; Koć-Januchta et al., 2019), can have an impact on the learning process. Regarding the construction of multimedia scaffolds, these may not only be composed of descriptive and depictive representations (Ainsworth, 2006), but also of verbal audio tracks or symbolic texts in combination with static and dynamic images (among others Nitz et al., 2014). For a targeted, effective as well as preferably individualized use of technology-enhanced scaffolds, multimedia design principles based on CLT and the theory of multimedia learning (Mayer, 2014) should be resorted to minimize ECL and create free capacities in the working memory. Therefore, the use of multimedia scaffolds in the complex problem-solving process of inquiry-based learning (Figure 1: E+T) along with learner characteristics as causal factors of CL should be elicited.

### Learners’ (L) Characteristics as Causal Factor

Learner-specific characteristics are elementary in the context of CL (Figure 1) and thus also in the long-term memorization of new knowledge elements (among others Choi et al., 2014). Besides motivational and affective factors, interest (Baars et al., 2017) or fun (van de Weijer-Bergsma and van der Ven, 2021), the influence of cognitive characteristics, such as prior knowledge (Kalyuga et al., 2003; Cierniak et al., 2009a; Kalyuga, 2013), in relation to CL has been widely investigated. Interactions with the theory of multimedia learning as well as distinction of further cognitive characteristics aiming at cognitive abilities in relation to representation-based information processes, such as the development of reading skills, spatial ability, or cognitive-verbal and visual abilities, were examined so far only little in relation to CL or with unclear results (among others Ho et al., 2014; Chen et al., 2018; Lehmann and Seufert, 2020). However, based on theoretical frameworks of multimedia learning (Moreno, 2010; Mayer, 2014), human cognitive architecture (Paas and van Merriënboer, 2020), and empirical studies of individual cognitive abilities, assumptions can be made that identify a relationship between specific cognitive, representation-based, abilities, the learning material, and the resulting CL. In the following, specific (cognitive) characteristics are identified and elicited in terms of a possible causal factor in connection with CL and their possible of measurement within the present study.

#### Prior Knowledge

Studies investigating prior knowledge have shown that it significantly influences learning performance (Kalyuga, 2013; Chen et al., 2018; Richter et al., 2018; Seufert, 2019). Based on the cognitive architecture of knowledge processing and memorization, increased prior knowledge contributes to chunking (new) information and minimizing element interaction in the working memory (Pollock et al., 2002; Kalyuga, 2011). However, this is only correct up to a certain level of prior knowledge and dependent on the design of learning materials. For example, very detailed worked-out examples can minimize the learning outcome for students with a high level of prior knowledge, as their own individual knowledge construction is inhibited by the content specifications, whereas learners with a low level of prior knowledge can particularly benefit from these scaffolds (expertise-reversal effect: Kalyuga, 2013; Chen et al., 2016). In the context of inquiry-based learning, studies show that novices benefit less from individual knowledge construction than experts because they are missing prior experience or concrete information about the individual knowledge facets (e.g., Shrager and Siegler, 1998; Siegler, 2005; Kendeou and van den Broek, 2007). Reasons for this could be associated with high levels of CL and a consequent reduction of GCL, because the resources of the working memory for processing the learning content are exceeded (element interactivity, see “Introduction”). If the complexity of the learning task increases, explicit instruction is beneficial for long-term retention (Chen et al., 2016, 2018).

#### Cognitive Abilities

Along with the investigation of instructional forms and its pros and cons for specific learners, different correlations regarding CL can also be identified (e.g., Mayer and Massa, 2003). In addition to prior knowledge, spatial ability is increasingly a focus of investigation (e.g., Quaiser-Pohl et al., 2001). Basically, a positive correlation can be found between the expression of spatial ability and learning performance (Höffler, 2010). Regarding dynamic and static representations, such as animations or static images, spatial ability can act like a compensator according to the ability-as-compensator hypothesis (e.g., Höffler, 2010; Münzer, 2012; Sanchez and Wiley, 2014), but only if this ability is also actively needed for processing the learning task (Höffler and Leutner, 2011). Hence, it can be assumed that the higher spatial ability the better the learning performance when dealing with static images, whereas when learning with animated images, lower spatial ability may contribute to deeper understanding (e.g., Huk, 2006). Further determinants of how individual learning abilities are influenced can be identified using Jäger’s (1984) intelligence model. The recognition of patterns, the agreement of similarities, or other linguistic as well as visual contexts allow conclusions to be drawn about logical comprehension as well as verbal and visual thinking skills. A few studies show that here, the expression is also related to learning performance (Kuhn et al., 1988; Kaiser and Mayer, 2019). Along with verbal reasoning skills, reading skills can have an impact on individual learning performance, especially for text-based instructional materials (Plass and Homer, 2002; Plass et al., 2003; Jäger et al., 2017). In connection with the preference for specific, material-related instructional formats (see “Learning preference/Representation Preference”), reading skills can also have an influence on the processing of the task or the provided material (Peterson et al., 2015; Lehmann and Seufert, 2020). On the one hand, reading skills are also closely related to cognitive (verbal) skills. On the other hand, the expression of reading skills can have an influence on information processing especially when learning with text-based (monomodal) materials (Schneider et al., 2017).

#### Learning Preference/Representation Preference

Preferences with respect to a specific instructional material, modality, or representation fundamentally describe a person’s preferences to do or use something in a certain way than in another way (Kürschner et al., 2005; Lehmann and Seufert, 2020). The optional, student-specific selection of a learning material according to an individual’s preference, may result in positive effects in terms of, for example, motivation to complete the task (in reference to the self-regulation: Rheinberg et al., 2000; Baars et al., 2017). Mayer and Massa (2003) describe learning preference in (multimedia) learning situations as, e.g., visual or verbal learners showing a preference for a certain multimedia representation/instruction (representation preference). So, in specific learning situations, learners often prefer one multimedia representation for learning instruction (e.g., image and audio vs. image-text) over others (Mayer and Massa, 2003; Choi and Sadar, 2011). In the study of Lehmann and Seufert (2020), it was investigated whether the preference of a modality has an influence on learning performance and CL. Although this study did not examine multimedia learning materials, but only used monomodal text as visual or auditory stimuli, the results suggest a possible relationship between learning preference and CL. Their findings provide insights into the relationships between the (learning-) preference, the use of the respective instructional material (only modality), and the expression of CL. Thus, learners who associated their preferred modality with visual texts benefited and reported a lower ECL than those who did not use a modality matching their indicated preference (Lehmann and Seufert, 2020).

### Aims of the Study, Research Question, Assumptions, & Hypotheses

The aim of this work is to validate, along the Argument-Based Approach to Validation by Kane (2013) and Standards for Educational and Psychological Testing (AERA et al., 2014), a subjective instrument for students of the 9th and 11th grade for measuring the three different types of CL (ECL, ICL, and GCL):

(a) Based on the theoretical construct according to Choi et al. (2014), relationships between causal and assessment factors can be identified, which need to be tested in order to validate a subjective measurement instrument for the three different types of CL (Figure 1). In particular, the assessment of cognitive load resulting from instructional design (ECL; multimedia scaffolds) and its interactions/relationships with causal factors (representation preference; cognitive abilities) is of great interest for the present study.

(b) A possible convergence between the subjective measurement of CL with an objective instrument (eye-tracking metrics) will be checked. Both measures the load during the use of multimedia scaffolds and the related task for the *planning* phase of the inquiry process. Furthermore, a deeper investigation of possible correlations and influences of causal factors (cognitive-verbal and visual abilities, reading skills, and representation preference) through different analysis steps will be proofed.

According to this, the following overall, methodical research question will be investigated: *Do subjective ratings of cognitive load (ECL, ICL, and GCL) correlate with other measures or indicators of CL?* To investigate the research question, we made different validation assumptions that are differentiated *via* hypotheses and empirically tested in two studies. However, only assumptions and hypotheses are listed where we expect significant correlations, influences, and effects in respect to the CL derived from the theory.

*Assumption 1) The subjective measurement has an internal consistency and separates the relevant subscales – ECL, GCL, and ICL*.

**H1.1:** By adapting the items from previously tested instruments of CL (Cierniak et al., 2009b; Leppink et al., 2013; Klepsch et al., 2017), the three subscales ECL, GCL, and ICL can be identified both verbally and by statistical characteristics (internal structure and internal consistency).

**H1.2:** The CL subscales show positive and negative correlations to each other. The ECL scale correlates negatively with the GCL; the ICL scale correlates positively with the GCL (Leppink et al., 2013; Klepsch et al., 2017).

*Assumption 2) The subjective instrument leads to different cognitive load levels for the respective students in grades 9 and 11*.

**H2.1:** Higher prior knowledge, which students should have in grade 11, should result in lower CL than that of grade 9 students (Kalyuga, 2013; Chen et al., 2018; Richter et al., 2018; Seufert, 2019).

*Assumption 3) Students’ specific scores in the causal factors – cognitive-verbal and visual abilities, reading skills, spatial ability, and representation preference affect the individual CL (ECL, GCL, and ICL)*.

**H3.1:** There is a correlation between cognitive-verbal ability scales and CL, especially for text-based (monomodal) scaffolds (Kuhn et al., 1988; Mayer and Massa, 2003; Lehmann and Seufert, 2020).

**H3.2:** Cognitive-visual ability correlates negatively with ECL and positively with GCL (Jäger, 1984; Jäger et al., 2017).

**H3.3:** For the selected text-based (monomodal) scaffolds, reading-skill scores correlate negatively with ECL scores and positively with GCL scores (Plass et al., 2003; Lehmann and Seufert, 2020).

**H3.4:** Spatial ability may have an impact on CL when using static scaffolds (static image). It is possible that a high spatial ability minimizes the ECL and contributes to the increase of the GCL (ability-as-compensator hypothesis; Höffler, 2010; Münzer, 2012).

**H3.5:** The individual representation preference will condition the choice of a multimedia digital scaffold. The choice and the resulting possible fit between representation preference and use of the scaffold will be reflected in a low average ECL (Kürschner et al., 2005; Lehmann and Seufert, 2020).

*Assumption 4) As an objective instrument, visual attention in terms of specific eye-tracking metrics (total fixation count; mean fixation duration; and total fixation count) shows a high convergence with the levels of subjectively measured cognitive load (ECL, ICL, and GCL)*.

**H4.1:** There is a significant correlation between the total fixation count and the level of CL. The total fixation count predicts the ECL (Zu et al., 2020).

**H4.2:** The mean fixation duration and total fixation duration are significantly related to the level of CL. The mean fixation duration and total fixation duration predict the ECL (Zu et al., 2020).

## Materials and Methods

### General Design of the Study I and II

To test the subjective measurement of CL, two studies were conducted within FLOX, a teaching-learning laboratory of the University of Kassel. In both studies, the intervention, measurement times, and tasks were identical. Thus, students experimented in small groups (three students) along the scientific inquiry process on a selected topic of metabolic physiology (see “Environment (E) & Task (T) as causal factor – scaffolding & inquiry learning”; “Procedure”). The experiment as well as the experimental components, the scaffolds, the instructional material, and group size did not differ in both sub-studies. However, the difference was in the playback device of the scaffolds and in the time duration of the experiment (see “Procedure”; “Scaffolds & Task”). Whereas in study I, the scaffolds were provided to the participants *via* a tablet, in study II, the students watched their scaffold on a computer. In contrast to study I, which was performed with an entire class (environment), study II could only be conducted with three students at a time (in a similar total duration of the laboratory day in study I), and therefore, the experiment designed by the students was not performed in study II. The total duration of the laboratory day was 3–4h.

### Participants

Participants in both studies (*n*=250, 53.2% female) were 9th (*n*=142) and 11th (*n*=108) grade students from schools in Kassel, Germany (see Table 1 for details). Divided into the two sub-studies, study I involved 181 students (9th grade: *n*=111; 11th grade: *n*=70), whereas the second study employed a total of 69 students (9th grade: *n*=31; 11th grade: *n*=38). Both student groups and their legal guardians were given written and verbal information about the study. Based on this, both parties had to give their written consent to participate in the study. This was voluntary and all student data during the intervention were recorded anonymously.

**Table 1:** Demographic data from the studies.

		*Total sample (Study I+II)*			*Study I*			*Study II*		
		*M*	*SD*	*n*	*M*	*SD*	*n*	*M*	*SD*	*n*
**Age, years**		15.82	1.10	250	15.77	1.13	181	15.95	1.05	69
**Sex, % female**		53.2		250	48.1		181	66.7		69
**Grade**	**9th**			142			111			31
	**11th**			108			70			38

### Procedure

One week before each experimental-laboratory day, a pre-instruction including a pre-assessment was conducted within regular school hours. At this first measurement point, the students’ demographic data, their cognitive abilities (Cognitive Ability Test: Heller and Perleth, 2000), and reading skills (Reading Speed and Reading Comprehension: Schneider et al., 2017) were assessed in a written form (Table 2). On the laboratory day, small groups of three students were first randomly assigned. Following this, the general procedure of the laboratory day was explained with the participants. In addition, the individual scaffolds (static image-text=image-text; static image-audio=image-audio, moving image-text=animation; and moving image-audio=video) were introduced by content-remote examples since the subsequent selection of the preferred scaffold was made independently by the students. After examining the phenomena “A mushroom in the pizza dough” in a video jointly in the classroom/laboratory (study 1) or in groups (study 2), the independent, experimental work along the inquiry-based learning process within the small groups followed. After observing the phenomenon, the students were first invited to independently pose a research question (for example: *What influence does temperature have on the activity of yeast?*). Afterward, the groups formulated two well-founded hypotheses in line with the research question, which they would like to test in their experiment. The hypothesis formulation was followed by the *planning* phase of the experiment, which is also the intervention phase. At the beginning of the intervention, the students were initially asked to continue working individually. Along their protocol, in which the students documented all results of the experimentation phases, they were first instructed to individually select one of the four provided scaffolds. In study I, they viewed them on a tablet. In study 2, they used a computer with a stationary eye-tracking system (Tobii-Pro X3-120; 120HZ; <0.4°; 22-inch screen). Before each eye-tracking recording, a short technical briefing and an individual 9-point calibration were performed. To optimize the eye-tracking recording and to minimize any unnecessary distractions of the participants, some preparations were made, such as the light controlling of the eye-tracking areas and the separation of working areas (Kastaun et al., 2020; Kastaun and Meier, 2021). Directly after using the multimedia scaffolds, a subjective measurement of individual CL was integrated into their protocol. After completing the CL test, students had the task to operationalize the variables for their experiment before going into the group work again. Then, they developed a complete plan in the group to test one of their hypotheses. Afterward, the groups conducted their experiment together (study I) or used their planning anticipated videos that showed the experimental implementation and matching value tables (study II). After that, the students interpreted the results in reference to the research question and hypotheses.

**Table 2:** Descriptive data of used measurement of assessment and causal factors for the total sample.

	*M*	*SD*	*Cronbachs α*	*Items per Scale*	*References*
**ASSESSMENT FACTORS**					
Extraneous cognitive load (ECL)	2.17	1.01	0.891	5	Klepsch et al., 2017
Germane cognitive load (GCL)	4.04	1.06	0.814	3	Cierniak et al., 2009b
Intrinsic cognitive load (ICL)	3.50	1.34	0.696	2	Leppink et al., 2013
**CAUSAL FACTORS**					
***Cognitive abilities***					
Cognitive-visual ability (Figure classification, N01)	13.93	8.88	0.895	24	Heller and Perleth, 2000
Cognitive-visual ability (Figure Analogy, N02)	12.92	4.22	0.830	24
Spatial ability (Paper Folding, N03)	7.37	3.31	0.790	14
Cognitive-verbal ability (Word Analogy, V03)	7.5	2.6	0.700	19
***Reading skills***					
Reading comprehension	46.75	26.26	0.82	47	Schneider et al., 2017
Reading speed	41.62	28.36	0.80		
Reading accuracy	64.72	25.32	0.82	47	

### Scaffolds & Task

**Figure 2:**
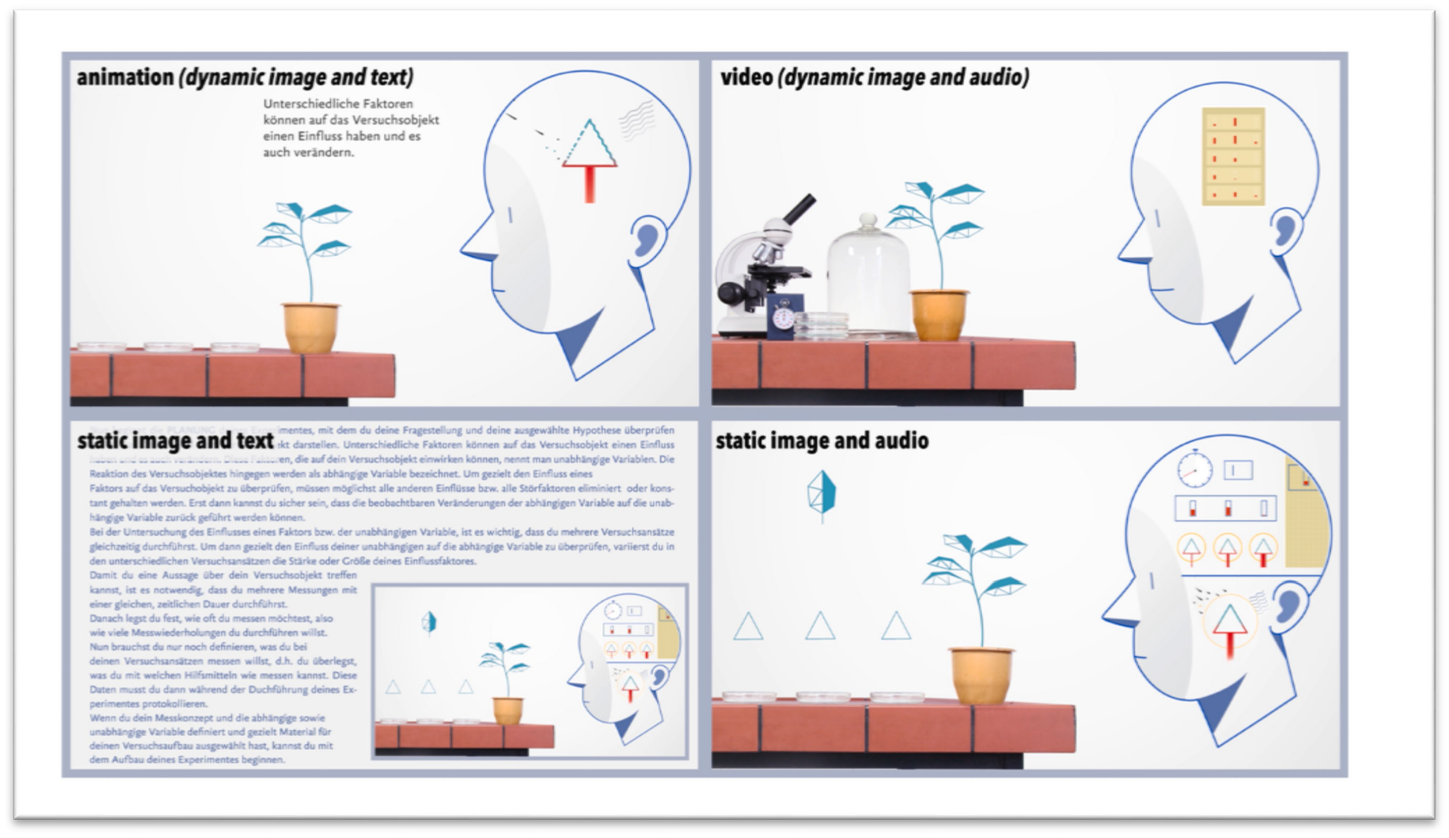
Image sections of the scaffolds in the different multimedia representation combinations.

In both studies, the students were required to choose one option from the same list of multimedia scaffolds (Figure 2). These differed in the combination and modality of their respective individual representations. Image-text and animation represent the monomodal scaffolds, whereas image-audio and video can be summarized as multimodal scaffolds with spoken text. The design of the graphics and the content of all scaffolds are identical and comparable according to cognitive-psychological principles (among others Mayer, 2014). The scaffolds have about the same time duration. The length is between 2.5 and 3min. The content consisted of written and spoken text as well as graphical representations to summarize the methodological skills/knowledge for the *planning* phase. Especially, the concept of variable operationalization and the creation of measurement series and associated measurement concept are focused without reference to any specific domain content (Meier and Kastaun, 2021). After the use of a scaffold and the cognitive load measurement, the students were asked to identify the dependent and independent variables (see “Environment (E) & Task (T) as causal factor – scaffolding & inquiry learning”). To do so, the students needed to use their prior knowledge as well as the information provided in the scaffold and transfer it to the specific subject context of the experiment. The use of the scaffold as well as the identification of the dependent and independent variables constitutes the task of the environment (Figure 1).

### Instruments & Methods

Different instruments and evaluation procedures were used to test the validation hypotheses. Table 2 lists the individual test instruments and associated scales.

#### Cognitive Load

The subjective instrument for measuring individual CL was adapted and composed of different items from validated test instruments (Cierniak et al., 2009b; Leppink et al., 2013; Klepsch et al., 2017; see Supplementary Table A). With the aim to measure the different load categories, ECL, GCL, and ICL, during learning with multimedia scaffolds, the paper-based questionnaire initially consisted of 15 different items (ECL=7 items; GCL=4 items; and ICL=4 items). These items were composed and adapted in such a way that they can be independently reflected upon and assessed by 9th and 11th grade students and have a primary relationship to the scaffolds. In addition, the subjective instrument is primarily intended to assess the CL based on the multimedia scaffolds. Therefore, the items were linguistically adapted and composed in this manner. Using a 6-point Likert scale from *strongly disagree (1)* to *strongly agree (6)*, the students’ self-reports were recorded in relation to the respective items. After the survey, we performed a Principal Component Analysis of the obtained datasets (using Varimax rotation) and the CL scales were formed by merging discrimination indices. The Cronbach’s *α*-values were determined to verify the internal consistency.

#### Reading Speed and Reading Comprehension Test

The Reading Speed and Reading Comprehension Test (Schneider et al., 2017) is an established test instrument in school diagnostics and is used for the differentiated determination of reading speed, reading accuracy, and reading comprehension from grades 5 to 12. It is a speed-power test in which a given text must be read as quickly and accurately as possible within a fixed time (6min). In the text, items are integrated at individual points, which must be used to complete the sentence by selecting the correct word. By analyzing the correctly crossed words, the reading accuracy across the text as well as the reading comprehension can be evaluated by a point system. To record reading speed, the number of all words read within the prescribed time was counted and evaluated using an evaluation scale.

#### Cognitive Ability Test

The Cognitive Ability Test (Heller and Perleth, 2000) is also an established test instrument in school diagnostics. It is used for the differential measurement of cognitive ability dimensions that are particularly relevant for learning at school. Scales of cognitive-verbal (V03) and visual abilities (N01, N02) as well as spatial ability (N03) were used from this. The individual instruments primarily aim to measure differential content-based, processing capacity. The Cognitive Ability Test is also a speed-power test. Within a prescribed time (8min), participants had to solve the individual test items of the respective scales. The visual, series tasks (N01), visual, and verbal relation tasks (N02 & V03) as well as paper-folding tasks measuring the spatial ability (N03: paper-folding test) were analyzed by an evaluation scale (correct/wrong), and the expressions of the individual constructs were identified by the formation of total scores.

#### Representation Preference

Representation preference was generated by an independent and free choice of the scaffold. The evaluation is generated *via* a frequency analyses of the individual usage behavior.

#### Gaze Behavior

Within study II (*n*=69), students’ gaze data were recorded using a stationary eye-tracking system. Using the software Tobii-Pro Studio, all students with a poor calibration were eliminated from the sample. In this work, we analyzed the learning stimuli as a whole, i.e., there was a single AOI that covered all information provided in the learning material. Afterward, the total fixation count, mean fixation duration, and total fixation duration were extracted using the software. Since the scaffolds have different characteristics in information retrieval and processing, the data were first *z*-standardized.

### Data Analysis

In the following analysis, we excluded datasets that showed missing values or too large measurement inaccuracies (outliers and dropout: *n*=23) or calibration errors (eye-tracking datasets, *n*=4; Gaze Sample under 65%) in the main intervention. Measurement data from the Reading Speed and Reading Comprehension Test and the Cognitive Ability Test may have different samples to the overall or sub-study as these datasets are partially incomplete. Nevertheless, these subjects were included in the main analysis regarding representation preference and CL since the complete datasets were available. The IBM SPSS Statistic 27 software was used for the statistical analyses.

To investigate the validity assumptions and research hypotheses, correlation and regression analyses as well as methods of inferential statistics (*t*-test and ANOVA) were applied for both studies. First, the internal consistency was examined in more detail by factor analytic procedures and correlation analyses of the individual scales of the CL test (validity assumption 1). To test the second validity assumption, different *t*-tests were conducted to analyze the assumed differences in the types of cognitive load (ECL; GCL; and ICL) between the two grade levels (9th and 11th grade). In addition, group differences (ANOVA) between the selected scaffolds (representation preference) and the expressions of the ECLs, GCLs, and ICLs were analyzed for each grade in order to exclude possible variance due to the different multimedia representations. Along the third validity assumption, correlations with the individual scales of the Reading Speed and Reading Comprehension Test as well as the Cognitive Ability Test in relation to the individual expressions of CL were conducted. Multiple regression analyses were used to further examine these correlations to identify predictors of CL during the use of the various scaffolds. No violations of the predictors were identified for any of the regressions. The Durbin-Watson statistics for all regressions ranged from 1 to 3 and no autocorrelations between variables were present (VIF<10). Furthermore, possible correlations (multiple regressions with categorical variables) and differences (*t*-test) between representation preference and the expression of representation preference were investigated. The quantitative data from the second study were used to test the fourth validity assumption. For this purpose, the *z*-standardized eye-tracking metrics (total fixation count; mean fixation duration; and total fixation count) were correlated with the individual expressions of CL.

## Results

### Validity Assumption I

We performed a Principal Component Analysis (Varimax rotation) to extract the most important independent factors (KMO=0.751; the Bartlett Test was highly significant; *p*<0.001). Kaiser’s criteria and the scree-plot yielded empirical justification for retaining three components (value ≥1) which accounted for 65.76% of the total variances. From these dimension-related items, the individual scales of CL were composed and reduced from an initial set of 15 items to 10 (ECL=5 items, GCL =3 items, and ICL=2 items; see Supplementary Table A). Items were excluded that either had too low factor loadings (<0.3) or loaded too high on another factor that was not integrated in the construct. These resulting 10 items and three scales were checked for internal consistency using Cronbach’s *α* (Table 2) and correlation analyses of the individual subscales (Table 3). The examination of the internal consistency by the Cronbach’s *α*-values of the individual subscales and the total scale for the CL shows satisfactory characteristic values (Table 2). As expected, statistically significant correlations between the ECL and GCL (*p*<0.001) and ECL and ICL (*p*<0.001) were found. However, the scales of the GCL and ICL did not show a statistically significant relationship (*p*=0.61; Table 3).

**Table 3:** Parallel-test reliability *via* Pearson-correlations (*n*=250).

		GCL	ICL
ECL	*r*	−0.38	0.23
	*p*	**0.00**	**0.00**
GCL	*r*		0.03
	*p*		0.61

*p* < 0.05.

### Validity Assumption II

To test the second validity assumption stating that CL decreases in progressive, school-based education due to an increase in methodological skills, group comparisons (*n*=250) were made regarding the CL between 9th and 11th grade students. The groups differed statistically significantly in all scales of CL (Table 4). A large effect was shown according to Cohen (1988) regarding the ICLs (*d*=0.822). The 11th grade students rated their intrinsic CL about one value lower than the 9th grade students. Medium effects were identified in the ECLs (*d*=0.552). Regarding GCL, the groups differed statistically significantly but only with a small effect size (*d*=0.295). Thus, the GCL conducive to learning increases as expected in the higher grade level by a value of 0.3. To support the results and to strengthen the link to prior knowledge as an influencing variable, single factor ANOVAs with Bonferroni-correction were conducted on the expression of the individual types of CL and the representation preference (choice of scaffold). The results show that only the ECL (*F*(3,3)=3.359, *p*=0.021, $\eta_{p}^{2}$=0.068) in the 9th grade differed significantly between the learners who chose an image-text combination (*n*=37; *M*=2.82; *SD*=1.08) and those who, in contrast, chose a video as a scaffold (*n*=56; *M*=2.13; *SD*=1.04). Furthermore, there were significant differences within the 11th grade with respect to the GCL (*F*(3,3)=3.187, *p*=0.027, $\eta_{p}^{2}$=0.085) between the groups of animations (*n*=29; *M*=4.56; *SD*=0.86) and picture text (*n*=38; *M*=3.86; *SD*=0.97). For the ICL and between the other groups regarding the ECL and GCL, no significant differences could be identified (see Supplementary Material).

**Table 4:** Differences between 9th and 11th grade in cognitive load (*t*-test, *n*=250).

	Grade 9th (*n*=142)		Grade 11th (*n*=108)		
	*M*	*SD*	*M*	*SD*	
ECL	2.41	1.06	1.87	0.87	(95%−CI[0.29, 0.78])/*t*(248)=4.26, *p*<0.001, *d*=0.552, *r*= 0.26
GCL	3.90	1.07	4.22	1.03	(95%−CI[−0.57, −0.04])/*t*(248)=−2.27, *p*=0.024, *d*=−0.295, *r*=0.14
ICL	3.94	1.27	2.92	1.22	(95%−CI[0.70, 1.33])/*t*(248)=6.36, *p*<0.001, *d*=0.822, *r*=0.38

### Validity Assumption III

To test the third validity assumption, correlation and regression analyses were conducted and evaluated for the total sample (*n*=250; Tables 5–11) and the subsamples (study I: *n*=181; study II: *n*=69; see Supplementary Material). Negative correlations between cognitive-verbal abilities (V03) and the expressions of the ECL (*r*=0.17; Table 5) as well as positive ones with the GCL (*r*=0.14, Table 5) were found. Moreover, showing a small correlation coefficient, the expressions of reading speed (*r*=0.19) and reading comprehension (*r*=0.16) are positively related to the scale of the GCL’s. Regarding the cognitive-visual and spatial scales (N01, N02, and N03) as well as the reading accuracy scale, no other statistically significant correlations could be identified (Table 5). Correlation analyses within the sample of the first study (*n*=181, see Supplementary Material) further revealed statistically significant correlations between the ICL and the expression of reading comprehension (*r*=0.21). Moreover, significant correlations between reading speed (*r*=0.21) and cognitive-visual ability (N01; *r*=0.16) and the expression of the GCL were identified. However, for the second study (*n*=69, see Supplementary Material), only one significant correlation could be found. Cognitive-visual ability (N02) correlated significantly negatively with the ICL scale (*r*=0.29). Apart from that, multiple linear regressions were used to examine the cognitive abilities (N01, N02, N03, and V03) and reading skill scales as possible predictor variables for CL in the use of multimedia scaffolds. Regarding the reading-skill scales for reading comprehension, reading accuracy and reading speed have no influence on the individual CL (see Supplementary Material). In contrast, the cognitive-verbal and visual abilities (V03, N01, and N02) scales showed a significant influence on the ECL (Table 6), GCL (Table 7), and ICL (Table 8) within the total sample. Multiple linear regressions with categorical variables were performed to investigate the influence of representation preference on the different types of CL. The results show a significant difference regarding the expression of the ECL and GCL between the learners who used scaffolds with static image and text and those who learned with the video (Tables 10, 11). The ECL of students who learned with static images and text was significantly higher than for the video users (*p*=0.008), whereas GCL decreased significantly (*p*=0.037). As expected from the validity assumption III, the representation preference that is equivalent to the voluntary choice of the scaffold had no further significant influence on the expression of CL (see Supplementary Material and Tables 10, 11). For an in-depth analysis of possible correlations or differences between representation preference and the expression of reading skills, cognitive abilities and CL, group differences between those who used multimodal and those who used monomodal scaffolds in the *planning* phase were examined. Differences were found with respect to the expression of reading speed (95%-CI[0.31, 17.59]/*t*(167)=2.05, *p*=0.042, *d*=0.318) and ECL (95%-CI[0.01, 0.51]/*t*(247)=2.01, *p*=0.046, *d*=0.256, *r*=0.132). Accordingly, users of a monomodal scaffolds exhibited significantly higher reading speed as well as ECL than those who selected and used a multimedia one. Furthermore, no statistically significant differences between the multimedia and monomodal users could be identified (see Supplementary Material). In addition, group differences between good and poor readers were analyzed regarding the GCL, ECL, and ICL scores for the text-based (monomodal) scaffolds. Again, no significant differences between the individual groups and the expression of cognitive load were found (see Supplementary Material).

**Table 5:** Pearson correlation between causal and assessment factors for the total sample.

		Cognitive-verbal ability (V03)	Cognitive-visual abilities (N01/02)		Spatial ability (N03)	Reading comprehension	Reading speed	Reading accuracy
ECL	*r*	−0.167	0.066	−0.085	−0.055	−0.112	−0.111	0.023
	*p*	**0.013**	0.323	0.202	0.409	0.143	0.148	0.766
GCL	*r*	0.143	−0.111	−0.001	0.067	0.163	0.190	0.044
	*p*	**0.034**	0.095	0.985	0.320	**0.034**	**0.013**	0.571
ICL	*r*	−0.062	0.118	−0.125	−0.082	−0.107	−0.125	−0.025
	*p*	0.359	0.079	0.063	0.226	0.164	0.102	0.744

*p* < 0.05.

**Table 6:** Multiple regression with ECL and cognitive abilities (*n*=222).

*Coefficient*	*B*	*SE(B)*	*BETA*	*P*	*VIF*
(Constant)	2.460	0.300			
V03	−0.068	0.028	−0.172	**0.016**	1.155
N03	−0.007	0.025	−0.024	0.765	1.446
N02	−0.020	0.020	−0.083	0.310	1.515
N01	0.040	0.020	0.152	**0.046**	1.315

*R*^2^=0.047, Durbin-Watson-Statistic=1.870. *p* < 0.05.

**Table 7:** Multiple regression with GCL and cognitive abilities (*n*=222).

*Coefficient*	*B*	*SE(B)*	*BETA*	*P*	*VIF*
(Constant)	4.081	0.314			
V03	0.069	0.029	0.168	**0.019**	1.155
N03	0.033	0.026	0.101	0.203	1.446
N02	−0.005	0.021	−0.021	0.798	1.515
N01	−0.052	0.021	−0.189	**0.013**	1.315

*R*^2^=0.052, Durbin-Watson-Statistic=1.677. *p* < 0.05.

**Table 8:** Multiple regression with ICL und cognitive abilities (*n*=222).

*Coefficient*	*B*	*SE(B)*	*BETA*	*P*	*VIF*
(Constant)	3.471	0.397			
V03	−0.024	0.037	−0.046	0.516	1.144
N03	−0.033	0.032	−0.080	0.314	1.435
N02	−0.052	0.026	−0.162	**0.048**	1.509
N01	0.079	0.026	0.228	**0.003**	1.310

*R*^2^=0.055, Durbin-Watson-Statistic=2.133. *p* < 0.05.

**Table 9:** Selection frequencies of scaffolds.

*Scaffolds*	*Selection frequencies*					
	*Total sample (n=249)*		*study I (n=180)*		*study II (n=69)*	
	*n_j_*	*h_j_*	*n_j_*	*h_j_*	*n_j_*	*h_j_*
image-text	75	30.1	53	29.4	22	31.9
image-audio	20	8.0	15	8.3	5	7.2
animation	62	24.9	46	25.6	16	23.2
video	92	36.9	66	36.7	26	37.7

*n*_*j*=_ absolute frequency=sum of used scaffolds in the respective format; *h_j_*=relative frequency (in percent)=relative share of the selected scaffolds in relation to the total number of scaffolds used.

**Table 10:** Multiple regression between ECL and representation preference (*n*=250).

*Coefficient*	*B*	*SE(B)*	*BETA*	*P*	*VIF*
(Constant)	1.951	0.105			
image-text	0.416	0.156	0.188	**0.008**	1.268
image-audio	0.449	0.248	0.120	0.072	1.120
animation	0.416	0.165	0.165	0.138	1.257

*R*^2^=0.033, Durbin-Watson-Statistic=1.939. *p* < 0.05.

**Table 11:** Multiple regression between GCL and representation preference (*n*=250).

*Coefficient*	*B*	*SE(B)*	*BETA*	*P*	*VIF*
(Constant)	4.170	0.110			
image-text	−0.346	0.165	−0.149	**0.037**	1.268
image-audio	−0.270	0.261	−0.069	0.302	1.120
animation	−0.009	0.174	−0.004	0.959	1.257

*R*^2^=0.023, Durbin-Watson-Statistic=1.873. *p* < 0.05.

### Validity Assumption IV

To test the fourth validity assumption, correlations (Table 12) were first calculated between the z-standardized eye-tracking metrics and the expressions of CL. Within the sample (*n*=69), a significant correlation between the total fixation count and the expression of ECL was found (*r*=0.247). Although no other significant correlations were evident, the correlation coefficient between the mean fixation duration and the expression of ECL, which is just above the significance level, also indicated a possible relationship. An examination of the total fixation count as a predictor for the individual CL revealed no significant results for GCL and ICL. In contrast, the total fixation count showed a significant effect on ECL (Table 13).

**Table 12:** Pearson correlation with eye-tracking metrics and cognitive load scales (*n*=69).

		Total fixation count	Mean fixation duration	Total fixation duration
ECL	*r*	0.247	−0.233	0.065
	*p*	**0.041**	0.055	0.596
GCL	*r*	−0.079	0.169	0.089
	*p*	0.517	0.165	0.467
ICL	*r*	−0.020	0.140	0.056
	*p*	0.871	0.252	0.646

*p* < 0.05.

**Table 13:** Linear regression between ECL and total fixation count (*n*=69)

*Coefficient*	*B*	*SE(B)*	*BETA*	*P*	*VIF*
(Constant)	−0.118	0.103			
Total fixation count	0.216	0.04	0.247	**0.041**	1.000

*R*^2^=0.061, Durbin-Watson-Statistic=2.220. *p* < 0.05.

## Discussion

The aim of the study was to test a subjective instrument for measuring differential CL for 9th and 11th grade students across different validity assumptions. Hereby, possible relationships between causal factors (learner characteristics: e.g., cognitive, verbal and visual abilities, reading skills, and representation preference) and assessment factors of CL were investigated (Figure 1). Furthermore, an objective measurement method (eye-tracking: total fixation count, fixation duration, fixation, and mean fixation duration) was used. With the constant inclusion of the overarching methodological research question (see “Introduction”), different analyses were carried out for the respective assumptions (see “Aims of the Study, Research Question, Assumptions & Hypotheses”). In the following, the individual validity assumptions will be discussed and combined in a final concluding discussion in relation to the research question.

### Discussion of Validation Assumptions I–IV

The first assumption targets the internal consistency as well as significant correlations between the expected scales of the ECL, ICL, and GCL for validity testing. The constructed subjective measurement instrument is closely based on existing test items (Cierniak et al., 2009b; Leppink et al., 2013; Klepsch et al., 2017). Even with language and content adaptation, the evidence-based structure to the CL is confirmed in the dimensions of the ECLs, ICLs, and GCLs (H1.1). The examination of internal consistency shows satisfactory to very excellent Cronbachs-alpha values (see “Validity Assumption I”) and, except for the values from the ICL, coincides with the results from other studies (Klepsch et al., 2017). In this context, possible reasons can be identified in the linguistic construction as these items of the ICL (*for example: “I found the identification of the dependent and independent variables to be difficult.,”* see Supplementary Table A) did not directly refer to the use of scaffold but to the definition of the individual variables, and thus, to the complexity of variable operationalization (Leppink et al., 2013; Klepsch et al., 2017; Klepsch and Seufert, 2021). In contrast, the GCL and ECL items refer almost exclusively to the scaffolds and less to the task within the *planning* phase of the experiment (see Supplementary Table A). This might be one reason why the GCL and ECL scales are not significantly correlated with each other, in contrast to the ECL and ICL scales (Leppink et al., 2013; see “Introduction”). Furthermore, the results confirm the additive relationship between the ICL and ECL (see “Introduction”; Sweller et al., 2011).

An examination of possible differences between the two grades 9th and 11th regarding the expression of CL comprises the second validity assumption. The group comparisons show results in line with expectations that the expression of CL and its categories ECL, GCL, and ICL is less pronounced in 11th grade students than in 9th grade. This can be attributed to differently developed prior knowledge regarding the content, and it is likely to constitute a consolidation of the more developed self-regulation of the learners from 11th grade (among others Richter et al., 2018; Eitel et al., 2020; H2.1). Considering the group differences in the respective grade level regarding the used scaffold and the expression of the ECL, ICL, and GCL, the results do not only indicate a difference in subject-related knowledge. In contrast to the older 11th grade students, who did not differ significantly in ECL, significant differences appear among the younger students between those who used an image-text combination and those who chose a video (see “Validity Assumption II”). This suggests that either the instructions from the video-based scaffold were easier to relate to each other due to their auditory and visual layout (Paivio, 2006; Mayer, 2014) or that the learners tended to overestimate their own abilities in dealing with the image-text representation combination. The last is of course also strongly related to the subject-specific complexity as well as the formulation of the ECL items, since, for example, “*unclear language*” (see Supplementary Table A) can be understood ambiguously. Furthermore, the significant difference in grade 11 in relation to the GCL among the learners who chose an image-text combination or an animation can be interpreted in such a way that the students encountered a hurdle during the linking and extraction of the content from the scaffolds (see “Validity Assumption II”). The dynamic presentation of text and images in the animations may have minimized the mental interconnection of the information. Another explanation could be that the image-text combination supports repetitive reading and self-regulated comprehension due to the static presentation (Kastaun and Meier, 2021).

Regarding the third validity assumption that the individual expression of the cognitive-visual and verbal abilities, reading skills, spatial ability, and representation preference (causal factors) is related to the CL, a differential overall assessment emerges. In comparison with the results of the spatial ability, there are correlations to the other causal factors from which different explanatory approaches can be derived in relation to the overarching question regarding the validity test of the subjective test instrument (H3.4). Cognitive-verbal and visual abilities (N01, N02, and V03) as well as reading skills are included as variables in some studies on CL but are not further investigated in a differentiated analysis of CL (e.g., Cierniak et al., 2009b). Furthermore, different test instruments exist and can be used to measure this, which makes it difficult to transfer the results in respect to the measured construct (e.g., verbal reasoning ability; e.g., van de Weijer-Bergsma and van der Ven, 2021). Thus, it is currently not clear how cognitive abilities relate to the individual scales of CL. In the present study, relationships and influences of cognitive-verbal and visual abilities as well as influences on CL and its differentiated categories could be identified (Tables 5–11; H3.1; H3.2; and H3.3). Possible explanations can be found, among others, in the construction of the items and the item structure (see “Cognitive Ability Test,” relationship and series tasks) of the individual scales of the cognitive ability test used in this study (Heller and Perleth, 2000). These scales (N01, N02, and V03) measure content-based skills by determining logical thinking using content-based, here, visual and verbal examples *via* series/classification (N01) and ratio/analogy tasks (N02 & V03). According to Jäger et al.’s (2006) intelligence model, in which the scales measure verbal and visual abilities under a constant reference to the individual thinking ability, content-based cognitive abilities contribute to a person’s general intelligence. In terms of CL, this means that the correlations are not only due to students’ verbal or visual level, but also due to the ability to easily recognize links between different information. Furthermore, the results can also be explained by the construction of the scaffolds. The primary information carriers of the multimedia scaffolds are the verbal or textual representations, i.e., the visual representations also contribute to content development but without the verbal or text-based elements they do not explain the main content.

Considering the multimedia learning theory (Moreno, 2010; Mayer, 2014), which states that verbal and text-based information can be reorganized in working memory *via* images and sounds, high verbal cognitive reasoning ability can help minimize ECL and increase GCL. This is also evident from a closer look at the subsamples (see “Validity assumption III” and Supplementary Material). The explanatory approach, which implies that those with a higher expression of cognitive-verbal abilities benefit more than those who show an increased visual ability due to the design characteristics (among others Choi and Sadar, 2011), is supported by the significant negative correlations present in study I and II (see “Validity assumption III”) as well as by the findings of the multiple regression analyses. These show a strong to moderate effect of cognitive abilities on the individual load categories. In respect to the scaffolds used, cognitive-verbal abilities predicted low ECL and higher GCL, whereas cognitive-visual abilities scales did not predict any of the CL categories (see “Validity assumption III”).

Concerning the study of reading skills as a causal factor to CL, the results show that positive relationships exist between reading comprehension as well as reading speed and GCL. On the one hand, this can be attributed to the fact that cognitive-verbal reasoning skills are closely and positively related to the individual reading skills (Schneider et al., 2017). On the other hand, it shows that the reading process is an active construction activity (Artelt et al., 2007; Scheerer-Neumann, 2018). Thus, the significant correlations may result from the higher load based on the selected scaffold (monomodal) but also from the text-based self-assessment of the CL. Accordingly, the text-based test itself may have elicited a correlation between the GCL and reading comprehension or reading speed. Contrary to the identified correlations, the results of the multiple regression analyses do not show any significant influences of the reading skills on the three types of CL (see Supplementary Material). Further analyses between the use of the monomodal/multimodal scaffolds and the expression of cognitive load in relation to the reading skills also showed differentiated results. It is possible that the reading skills do not have a significant influence on the decision of the selected scaffold but still show advantages in the processing of the information (based on the modality) about the load.

The relationship between spatial ability and CL (H3.4), which has been supported and assumed by many studies (e.g., Höffler, 2010; Anvari et al., 2013), is not evident in the present study. As already stated by Höffler and Leutner (2011), among others, it also becomes clear with the present results that the spatial ability only has a significant influence on task performance if the task itself requires it. Consequently, it can be concluded that spatial awareness is not required for processing the information of the scaffolds used here.

Finally, the assumed correlations between the causal factor of representation preference and CL can be partially confirmed (H3.5). Independent of the selected scaffold, the ECL shows a very low expression (see “Procedure”). Multiple regression analyses confirm that the representation preference, i.e., the choice of scaffold, influences ECL and GCL. Thus, those students who show a preference for the image-text representation combination are more likely to experience significantly higher ECL than those who use the video. This is also evident in the GCL, which negatively predicts preference for image-text compared to video users. This can be attributed to the level of complexity of the text-based or auditory text (Leahy and Sweller, 2011; Lehmann and Seufert, 2020) and/or to the multimedia presentation mode of the verbal text in terms of simultaneous, dynamic visualizations (Moreno, 2010; Mayer, 2014) or due to the students’ failure to assess what kind of multimedia representation they could best handle in the situation (instruction).

The examination of a possible convergence between the subjective and objective instruments for CL (validity assumption IV) showed results partially confirming to expectations. The positive correlation (Table 12) and the significant influence (Table 13) of the absolute fixation count support the assumed convergence (H4.1; Zu et al., 2020). Zu et al. (2020) found a moderate negative correlation between the ECL and the mean fixation duration and a weak correlation between the total visit duration on an animation and the GCL. In our work, we found a weak negative correlation between the ECL and the mean fixation duration that was just above the significance level (*p*=0.55) and a significant positive correlation between total fixation count and the ECL. This implies that previously reported relationships between eye-tracking measures and cognitive load do not translate to our setting. The examination of other eye-tracking metrics, such as transitions between the AOIs, was not possible based on the material used here since possible selection and integration processes cannot be investigated unambiguously *via* visual attention when including auditory scaffolds.

### Limitations and Implications for Future Work

Along the individual validity assumptions based on the theoretical interpretation-use argumentation (Kane, 2013), arguments for a possible valid measurement *via* the test scores of the measurement instruments are used (causal & assessment factors; objective instruments), and their relationships to each other are identified. The central goal of validating a subjective measurement instrument for 9th and 11th grade students was partially achieved by confirming individual validity assumptions under study-specific conditions. The results support a valid measurement but also indicate that a validity test of a subjective measurement instrument for CL is associated with different difficulties. On the one hand, CL can be influenced by diverse causal factors in different ways (Figure 1; Choi et al., 2014), which is particularly related to the construction of the task and/or environment as well as its interaction with CL. On the other hand, a difficulty arises from the fact that there are no comprehensive, tested relationships between the individual subjective and objective measurement instruments for CL (assessment factors) in an evidence-based manner yet. Particularly regarding the cognitive abilities for visual and verbal reasoning, more detailed investigation is needed to determine whether these factors influence CL in a similar way as prior knowledge (expertise-reversal effect: Cierniak et al., 2009a; Kalyuga, 2013). Another limitation of our study is the missing inclusion of learning performance. Since the CLT assumes that CL also influences learning performance (see Figure 1), it would have been appropriate to include this in relation to the manifestations of cognitive load in relation to the causal factors. Due to our aims and the corroborating, temporally extensive design of the study, we were not able to consider learning performance. The focus was on the design of the scaffolds and the resulting cognitive load. In the future, this should be captured by valid instruments for the methodological skills/knowledge for the *planning* (content of scaffolds) to extend the analyzed relationships between causal and assessment factors. However, the results of this study demonstrate that performance-based (cognitive abilities and reading skills) and objective (eye-tracking metrics) instruments are linked to the subjectively obtained test scores on individual CL. Therefore, further analyses are needed in the future to examine the relationships between the individual causal factors in relation to different tasks and the resulting CL. However, not only the findings between the causal and assessment factors show further approaches of investigation, but also the subjective measurement instrument from its linguistic composition by itself. Thus, the results indicate that especially the linguistic construction of the items has a great influence on the actual measurability of the individual constructs. On the one hand, there is the difficulty of phrasing the items (especially GCL and ICL) in such a way that they reflect the construct to be measured (Wording, see “Introduction”). Above all, the measurement of the GCL must be considered with reservations. In addition to the term “knowledge,” the word “understanding” is used in the items. It is questionable whether students’ understanding and comprehension can actually be equated with GCL (Ayres, 2018), and how the corresponding items can be worded differently to express quantify GCL. On the other hand, there is the challenge of adapting the linguistic level to the learners in such a way that they can understand and assess it independently (Sweller et al., 2011; Klepsch and Seufert, 2021). In conjunction with the validation approach according to Kane (2013), which considers the generalizability of the test instrument as an elementary part of validity, the existing subjective test instruments should be examined in the future regarding a possible standardization with tested adaptation possibilities that fit the investigated setting (self-regulated learning or problem-based learning).

## Data Availability Statement

The raw data supporting the conclusions of this article will be made available by the authors, without undue reservation.

## Ethics Statement

Ethical review and approval was not required for the study on human participants in accordance with the local legislation and institutional requirements. Written informed consent to participate in this study was provided by the participants’ legal guardian/next of kin.

## Author Contributions

MM developed the basic idea for the present study and supervised the project. MK and MM contributed to the conception and design of the studies. MK developed the used material and questionnaires and led data collection for studies, analyzed all the data and interpreted it with the help of MM, SK, and JK, and was mainly responsible for writing the paper. SK and JK supported in the execution and analysis of the eye-tracking data. This article is part of the Ph.D. thesis of MK supervised by MM. All authors agreed to the final submitted version of the manuscript and agreed to be responsible for all aspects of the work, ensuring that questions regarding the accuracy or integrity of any part of the work were adequately investigated and resolved.

## Conflict of Interest

The authors declare that the research was conducted in the absence of any commercial or financial relationships that could be construed as a potential conflict of interest.

## Publisher’s Note

All claims expressed in this article are solely those of the authors and do not necessarily represent those of their affiliated organizations, or those of the publisher, the editors and the reviewers. Any product that may be evaluated in this article, or claim that may be made by its manufacturer, is not guaranteed or endorsed by the publisher.
