# Supplementary material for: Validation of Cognitive Load During Inquiry-Based Learning With Multimedia Scaffolds Using Subjective Measurement and Eye Movements
Source: Front Psychol. 2021 Aug 31;12:703857. doi: 10.3389/fpsyg.2021.703857 (PMC8438403; doi:10.3389/fpsyg.2021.703857)
Supplement: Supplementary file 1 [file Table_1.DOCX]

**Supplementary Material**

**Table A:** Subjective cognitive load measurement instrument (final version are items in black; rejected items after factor analysis in gray) with a *6-point Likert scale from (1) strongly disagree to (6) strongly agree.*

| ***Cognitive Load*** | ***(adapted) Items***  ***in german*** | ***(adapted) Items***  ***in english*** | ***Reference/s***  *(adapted to)* |
| --- | --- | --- | --- |
| **ICL** | Bei der Definition der abhängigen und unabhängigen Variable für das Experiment musste man viele Dinge im Kopf gleichzeitig bedenken. | In defining the dependent and independent variables for the experiment, one had to think of many things at the same time. | Klepsch et al., 2017 |
| **ICL** | Die Benennung der abhängigen und unabhängigen Variable empfand ich als sehr schwierig. | I found the naming of the dependent and independent variable very difficult. | Leppink et al., 2013 |
| **ICL** | Bei der Lernunterstützung zur Planung musste man viele Dinge im Kopf gleichzeitig bedenken. | In the scaffold for planning, I had to think of many things in your head at the same time. | Klepsch et al., 2017 |
| **ICL** | Die Inhalte der Lernunterstützung zur Planung empfand ich als sehr schwierig / komplex. | I found the content of the scaffold for planning to be very difficult / complex. | Leppink et al., 2013 |
| **ECL** | Die Texte die ich gelesen oder gehört habe in der Lernunterstützung zur Planung waren für mich sehr unklar. | The texts I read or heard in the scaffold for the planning phase were very unclear to me. | Leppink et al., 2013 |
| **ECL** | Die Kombination aus Bildern und gehörten oder gelesenen Texten in der Lernunterstützung zur Planung waren für mich sehr unklar. | The combination of pictures and heard or read texts in the scaffold for planning phase were very unclear to me. | Leppink et al., 2013 |
| **ECL** | Bei der Lernunterstützung zur Planung empfand ich es als mühsam, die wichtigsten Informationen zu erkennen. | In the scaffold for planning phase, I found it tedious to identify the most important information. | Klepsch et al., 2017; Leppink et al., 2013; Cierniack et al., 2009b |
| **ECL** | Bei der Lernunterstützung zur Planung ist es mir schwer gefallen, die Inhalte miteinander in Verbindung zu bringen. | In the scaffold for planning phase, I found it difficult to connect the content. | Klepsch et al., 2017; Leppink et al., 2013 |
| **ECL** | Die Erklärungen in der Lernunterstützung waren für mich voller unklarer Sprache. | The explanations in the scaffolds were full of unclear language for me. | Leppink et al., 2013 |
| **ECL** | Die Darstellung der Inhalte in der Lernunterstützung zur Planung finde ich ungünstig, um wirklich was zu lernen. | I find the presentation of the content in the scaffold for planning inconvenient to really learn anything. | Klepsch et al., 2017; Leppink et al., 2013 |
| **GCL** | Die Lernunterstützung enthielt Elemente, die mich unterstützten, die Inhalte zur Planung besser zu verstehen. | The scaffold included elements that helped me better understand the content for planning. | Klepsch et al., 2017 |
| **GCL** | Die Lernunterstützung hat mein Wissen und Verständnis zur Planung wirklich erweitert. | The scaffold has really enhanced my knowledge and understanding on planning phase. | Leppink et al., 2013 |
| **GCL** | Die Lernunterstützung hat mein Verständnis für die Planung wirklich verbessert. | The scaffold really improved my understanding of planning. | Leppink et al., 2013 |
| **GCL** | Ich habe mich angestrengt, bei der Lernunterstützung zur Planung mir nicht nur einzelne Dinge zu merken, sondern auch den Gesamtzusammenhang zu verstehen. | I made an effort in scaffold for planning to not only remember individual things, but also to understand the overall context. | Klepsch et al., 2017; Leppink et al., 2013 |

**Related to table 4 / Validity Assumption II**

Single factor ANOVA with post-hoc test (Bonferroni) of 9^th^ grade between the expressions of each cognitive load and the scaffold used (representation preference; *n* = 142)

|  | *Static image and text* | *Static image and audio* | *Animation* | *Video* | *F* |
| --- | --- | --- | --- | --- | --- |
| *ECL* | 2.82* | 2.43 | 2.4 | 2.13* | 3.359 |
| *ICL* | 3.79 | 4.18 | 3.95 | 3.96 | .359 |
| *GCL* | 3.39 | 3.91 | 3.80 | 4.04 | .562 |

**p* < 0.05

Single factor ANOVA with post-hoc test (Bonferroni) of 11^th^ grade between the expressions of each cognitive load and the scaffold used (representation preference; *n* = 108)

|  | *Static image and text* | *Static image and audio* | *Animation* | *Video* | *F* |
| --- | --- | --- | --- | --- | --- |
| *ECL* | 1.92 | 2.30 | 1.96 | 1.67 | 1.078 |
| *ICL* | 3.11 | 3.50 | 2.94 | 2.59 | 1.519 |
| *GCL* | 3.85* | 3.83 | 4.56* | 4.21 | 3.187 |

**p* < 0.05

**Related to table 5**

Pearson correlation between causal and assessment factors for study I (*n = 181)*.

|  |  | Cognitive verbal ability (V03) | Cognitive visual abilities  (N01/02) | | Spatial imagination  (N03) | Reading compre-hension | Reading speed | Reading accuracy |
| --- | --- | --- | --- | --- | --- | --- | --- | --- |
| ECL | *r* | -.168 | .122 | -.058 | -.064 | -.138 | -.116 | .032 |
|  | *p* | **.031** | .114 | .457 | .414 | .155 | .230 | .740 |
| GCL | *r* | .122 | -.159 | .005 | .069 | .166 | .185 | .073 |
|  | *p* | .118 | **.039** | .951 | .377 | .085 | .056 | .455 |
| ICL | *r* | -.107 | .124 | -.082 | -.077 | -.211 | -.209 | -.023 |
|  | *P* | .173 | .111 | .291 | .326 | **.029** | **.031** | .813 |

Pearson correlation between causal and assessment factors for study II (*n = 69)*.

|  |  | Cognitive verbal ability (V03) | Cognitive visual abilities  (N01/02) | | Spatial imagination  (N03) | Reading compre-hension | Reading speed | Reading accuracy |
| --- | --- | --- | --- | --- | --- | --- | --- | --- |
| ECL | *r* | -.088 | -.143 | -.139 | .049 | -.030 | -.087 | .011 |
|  | *p* | .517 | .289 | .303 | .719 | .813 | .496 | .930 |
| GCL | *r* | .162 | .089 | -.098 | -.009 | .133 | .194 | -.036 |
|  | *p* | .229 | .510 | .467 | .948 | .293 | .124 | .778 |
| ICL | *r* | .052 | .092 | -.298 | -.120 | .108 | .018 | -.038 |
|  | *p* | .702 | .494 | **.024** | .375 | .397 | .888 | .767 |

**Related to table 6-8**

Multiple regression with ECL und reading skills scales (*n = 168*).

| ***Coefficient*** | ***B*** | ***SE(B)*** | ***BETA*** | ***P*** | ***VIF*** |
| --- | --- | --- | --- | --- | --- |
| (Constant) | 2.542 | .282 |  |  |  |
| Reading comprehension | .002 | .008 | .049 | .819 | 7.507 |
| Reading speed | -.006 | .007 | -.150 | .433 | 6.088 |
| Reading accuracy | -.003 | .005 | -.062 | .618 | 2.574 |

R^2^ = .014

Durbin-Watson-Statistic = 1.876

Multiple regression with GCL und reading skills scales (*n = 168*).

| ***Coefficient*** | ***B*** | ***SE(B)*** | ***BETA*** | ***P*** | ***VIF*** |
| --- | --- | --- | --- | --- | --- |
| (Constant) | 3.612 |  |  |  |  |
| Reading comprehension | -.007 | .008 | -.185 | .381 | 7.507 |
| Reading speed | .011 | .007 | .315 | .098 | 6.088 |
| Reading accuracy | .004 | .005 | .105 | .394 | 2.574 |

R^2^ = .031

Durbin-Watson-Statistic = 1.781

Multiple regression with ICL und reading skills scales (*n = 167*).

| ***Coefficient*** | ***B*** | ***SE(B)*** | ***BETA*** | ***P*** | ***VIF*** |
| --- | --- | --- | --- | --- | --- |
| (Constant) | 3.624 | .379 |  |  |  |
| Reading comprehension | -.006 | .011 | -.155 | .589 | 7.450 |
| Reading speed | .000 | .009 | .004 | .982 | 6.129 |
| Reading accuracy | .002 | .007 | .037 | .771 | 2.592 |

R^2^ = .010

Durbin-Watson-Statistic = 1.907

Multiple regression between ICL and representation preference (*n* *= 250*).

| ***Coefficient*** | ***B*** | ***SE(B)*** | ***BETA*** | ***P*** | ***VIF*** |
| --- | --- | --- | --- | --- | --- |
| (Constant) | 3.423 | .141 |  |  |  |
| image-text | .030 | .210 | .010 | .885 | 1.270 |
| image-audio | .627 | .332 | .127 | .060 | 1.121 |
| animation | .052 | .222 | .017 | .817 | 1.258 |

R^2^ = .023

Durbin-Watson-Statistic = 1.873

**Related to table 9-11**

Differences between monomodal and multimodal scaffolds in reading skills, cognitive abilities and cognitive load (*n* = 250).

| ***reading skills scales*** | |
| --- | --- |
| Reading comprehension | 95%-CI[-1.12, 14.92] / *t*(166) = 1.69, *p* = .091, *d* = 0.264 |
| Reading speed | 95%-CI[.31, 17.59] / *t*(167) = 2.05, ***p* = .042,** *d* = 0.318 |
| Reading accuracy | 95%-CI[-4.45, 11.05] / *t*(169) = .841, *p* = .402, *d* = 0.130, *r* = 0.244 |
| ***cognitive abilities*** | |
| V03 | 95%-CI[-.54, .85] / *t*(219) = .45, *p* = .655, *d* = 0.061 |
| N03 | 95%-CI[-.33, 1.44] / *t*(221) = 1.24, *p* = .216, *d* = 0.168 |
| N02 | 95%-CI[-,93 11.05] / *t*(222) = .195, *p* = .846, *d* = 0.26 |
| N01 | 95%-CI[-1.05, 1.20] / *t*(222) = .135, *p* = .894, *d* = 0.18 |
| ***cognitive load*** | |
| ECL | 95%-CI[.01, .51] / *t*(247) = 2.01, ***p* = .046, *d* = .256, *r* = 0.132** |
| GCL | 95%-CI[-.41, .12] / *t*(247) = -1.07, *p* = .286, *d* = -.136 |
| ICL | 95%-CI[-.41, .27] / *t*(245) = -.42, *p* = .673, *d* = -.054 |

Differences between good and poor readers using monomodal scaffolds in ECL; GCL; ICL

| ***Reading comprehension (n_poorreader_ = 50 / n_goodreader_ = 48)*** | |
| --- | --- |
| ECL | 95%-CI[-.45, .42] / *t*(96) = -.06, *p* = .96 |
| GCL | 95%-CI[-.59, 1.17] / *t*(96) = -1.10, *p* = .275 |
| ICL | 95%-CI[-.33, .64] / *t*(96) = .631, *p* = .530 |
| ***Reading speed (n_poorreader_ = 57 / n_goodreader_ = 41)*** | |
| ECL | 95%-CI[-.17, .71] / *t*(96) = 1.23, *p* = .655, |
| GCL | 95%-CI[-.69, .08] / *t*(96) = -1.57, *p* = .120 |
| ICL | 95%-CI[-,43, .56] / *t*(96) = .272, *p* = .786 |
| ***Reading accuracy (n_poorreader_ = 28 / n_goodreader_ = 68)*** | |
| ECL | 95%-CI[-.80, .16] / *t*(94) = -1.32, *p* = .189 |
| GCL | 95%-CI[-.18, .67] / *t*(94) = 1.15, *p* = .254 |
| ICL | 95%-CI[-.17, .92] / *t*(94) = 1.38, *p* = .171 |
